# The Intracellular Loop 2 F328S Frizzled-4 Mutation Implicated in Familial Exudative Vitreoretinopathy Impairs Dishevelled Recruitment

**DOI:** 10.5334/1750-2187-10-6

**Published:** 2015-11-24

**Authors:** Milly S. Pau, Shujuan Gao, Craig C. Malbon, Hsien-yu Wang, Alexander C. Bertalovitz

**Affiliations:** Department of Physiology & Biophysics, School of Medicine, Health Sciences Center, Stony Brook University, Stony Brook, NY 11794-8661, USA; Department of Pharmacology, School of Medicine, Health Sciences Center, Stony Brook University, Stony Brook, NY 11794-8651, USA

**Keywords:** Frizzled, Frizzled-4, Norrin, Dishevelled, FEVR, Wnt

## Abstract

Familial exudative vitreoretinopathy (FEVR) is a disease state characterized by aberrant retinal angiogenesis. Norrin-induced activation of Frizzled-4 (Fz4) has a major role in regulating beta-catenin levels in the eye that, in turn, modulate the blood retina barrier (BRB). Here we gain insight on the basis of the pathology of a FEVR implicated F328S Fz4 mutant by study. The receptor exhibits a substantially reduced ability to activate Lef/Tcf-dependent transcription. This impaired activation correlates with a decreased ability to stabilize and recruit Dishevelled-2 (Dvl2) to the cell surface. Aromaticity at position 328 of the intracellular loop 2 (iloop2) is revealed similarly as a prerequisite for Dvl2 recruitment to the Fz4. This aromaticity at 328 enables normal Norrin-induced canonical activation. The corresponding position in iloop2 of other Frizzleds likely functions in Dvl recruitment.

## Introduction

The ten Frizzleds identified in the mammalian genome have critical roles in embryonic development, cell proliferation, angiogenesis, and other regulatory factors important for maintaining a homeostasis. Disruption of the receptors for Wnts, the Frizzleds, can result in cancer and other diseases [1]. A major determinant in embryonic and postnatal blood vessel regulation is Frizzled-4 [2]. Mutations in Fz4 have been implicated in retinal hypovascularization associated with familial exudative vitreoretinopathy and related retinopathies [3,4]. Mutations of the cognate ligand of Fz4, Norrin, of the coreceptor low-density lipoprotein receptor-related protein 5 (LRP5), or of transmembrane 4 superfamily member 12 (TSPAN12, which has a role in the multimerization of the signaling complex) are thought to promote FEVR [5,6,7,8]. Mutations in Zinc finger protein 408 (ZNF408) recently were demonstrated to promote FEVR. The mechanism by which these proteins modulates Norrin-mediated signal transduction remains unknown [9].

Fz4 and Norrin appear to homodimerize prior to interacting [10]. Three disulfide bonds between interacting Norrin molecules are required for maximal signal from Fz4. Both LRP5 and 6 have been demonstrated to augment Norrin as well as Wnt-induced Fz4 activation [2,11]. Following Norrin binding LRP is recruited to or stabilized at the receptor [10]. The constitutively-active beta-catenin destruction complex including GSK3 is disrupted enabling cytoplasmic beta-catenin accumulation and therefore activation of TCF/LEF transcription factors as beta-catenin accumulates into the nucleus [12].

Despite some reports that Fz4 can induce “non-canonical” or beta-catenin-independent signaling [13,14], current evidence suggests that reduced beta-catenin stabilization may correlate best with FEVR symptoms [15]. Deficiencies in both the BRB and the blood brain barrier (BBB) in mice lacking various components linked to Fz4-mediated signal transduction were substantially reversed by increasing the stabilization of beta-catenin. A Norrin variant has been implicated in FEVR, a mutation leading to over activation of canonical signaling [2]. Mutations in Fz4 that are associated with FEVR have been reported. Most of these mutations occur with high frequency in the putative ligand-binding, initial contact site, *i.e.*, the cysteine-rich domain [16,17]. A recent study of 15 Fz4 mutants with single-site mutations associated with FEVR indicated that nine of the variants had substantially reduced surface expression of the Frizzled-4. All but one of the Fz4 mutants exhibiting defective trafficking were associated with mutations on the extracellular surface. A variant with a mutation in the extracellular loop 3 proximal region of transmembrane domain 7 failed to traffic to the cell surface like the wild-type (WT) receptor [17].

Sequence analyses performed on multiple patients with FEVR have revealed Fz4 mutations [3,4]. Amongst the identified missense variants were the following: a tyrosine-to-histidine mutation at position 211 (Y211H) of the amino terminus; a threonine-to-arginine mutation at position 237 (T237R) of transmembrane domain 1; an arginine-to-cysteine mutation at position 253 (R253C) of intracellular loop 1; a phenylalanine-to-serine mutation at position 328 (F328S) of intracellular loop 2; and finally, an aspartate-to-asparagine mutation at position 470 (D470N) of extracellular loop 3.

We generated mutations in Fz4 and investigated the Y211H, T237R, R253C, F328S, and the D470N Fz4. Our goal was to gain insight into how these mutations alter Wnt signaling. Of these, we show the F328S Fz4 mutation yields a Frizzled with a decreased ability to activate the canonical pathway. Its inability appears to result from a failure to recruit and/or stabilize Dishevelled-2 (Dvl2) at the cell surface. The iloop2 of a mammalian Frizzled appears to be essential to Dvl recruitment and normal function.

## Materials and Methods

### Construction of plasmids

The mouse Frizzled-4 sequence was amplified by PCR, digested by restriction enzymes BamHI and HindIII and inserted into the prk5 vector (Addgene, Cambridge, MA) with the V5-tag at the amino terminus of Frizzled-4 between residues F37 and G38 after the signal peptide. V5-tagged mouse Frizzled-4 was used as a template to generate the mutants (Y211H, T237R, R253C, F328S, and D470N) using the QuikChange Site-Directed Mutagenesis Kit (Agilent, Santa Clara, CA). For the construction of Y211H, T237R, R253C, F328S and D470N, the following sets of forward and reverse primers were designed and employed to amplify the plasmid respectively:

5’-GCTACGATGCTGGCTTGCACAGCCGCTCAGCTAAG-3’ (forward primer),

5’-CTTAGCTGAGCGGCTGTGCAAGCCAGCATCGTAGC-3’ (reverse primer);

5’-GCTTCATCTCCACCACCTTCCGCGTGCTGACCTTCCTGATTG-3’ (forward primer),

5’-CAATCAGGAAGGTCAGCACGCGGAAGGTGGTGGAGATGAAGC-3’ (reverse primer);

5’- CAGGTTTTCTTACCCTGAGTGCCCCATCATATTTCTCAG-3’ (forward primer),

5’-CTGAGAAATATGATGGGGCACTCAGGGTAAGAAAACCTG-3’ (reverse primer);

5’-GACACTCACTTGGTCTTTGGCAGCCGGAC-3’ (forward primer),

5’-GTCCGGCTGCCAAAGACCAAGTGAGTGTC-3’ (reverse primer);

5’-GCACTCTTTCGATATTCTGCAAATGACTCAAACATGGCAGTTG-3’ (forward primer),

5’-CAACTGCCATGTTTGAGTCATTTGCAGAATATCGAAAGAGTGC-3’ (reverse primer).

F328A, F328W and F328Y mutations were created in the same manner using F328S as a template and with the following sets of forward and reverse primers designed and employed to amplify the plasmid respectively:

5’-GTTATTCTGACACTCACTTGGGCTTTGGCAGCCGGACTCAAGTG-3’ (forward primer),

5’-CACTTGAGTCCGGCTGCCAAAGCCCAAGTGAGTGTCAGAATAAC-3’ (reverse primer);

5’-CTGACACTCACTTGGTGGTTGGCAGCCGGACTCAAG-3’ (forward primer),

5’-CTTGAGTCCGGCTGCCAACCACCAAGTGAGTGTCAG-3’ (reverse primer);

5’-CTGACACTCACTTGGTATTTGGCAGCCGGACTC-3’ (forward primer),

5’-GAGTCCGGCTGCCAAATACCAAGTGAGTGTCAG-3’ (reverse primer).

The C-terminal green fluorescent protein (GFP)-tagged Dvl2 construct was generated by cloning the Human Dvl2 gene into the pEGFPN vector (Clontech, Mountain View, CA). All constructs were verified by DNA sequencing through the DNA Sequencing Facility (Stony Brook University, Stony Brook, NY).

### Cell culture

Human embryonic kidney (HEK293) and HeLa cells (obtained from ATCC, Manassas, VA) were grown in Dulbecco’s modified Eagle’s medium (Cellgro, Manassas, VA) supplemented with fetal bovine serum (10%) (Hyclone, South Logan, UT), penicillin (100 µg/ml) and streptomycin (100 µg/ml) (Corning, Manassas, VA) in a humidified atmosphere of 5% CO2 at 37°C.

### Lef/Tcf luciferase reporter assays

HEK293 cells cultured in gelatin-coated 96 well plates (Greiner Bio-One, Frickenhausen, Germany) were transfected at ~75% confluency with Lipofectamine 2000 (Life Technologies, Carlsbad, CA). Briefly, unless indicated in figure legends or the results section typical transfection conditions are as follows: functional assays included 0.5 ng of receptor, 1 ng of hLRP5 co-receptor, 10 ng of Super8xTOPFlash (M50) (a kind gift from Dr. Randall Moon, University of Washington, Seattle, WA) and pcDNA3.1 empty vector to a total of 50 ng plasmid DNA per well. Cells were stimulated with or without recombinant Norrin (200 ng/mL) (R&D Systems, Minneapolis, MN), Wnt3a (50 or 200 ng/mL) (R&D Systems, Minneapolis, MN) or LiCl (50 mM) for ~18 h 24 h post transfection. Cell were lysed in cell culture lysis buffer (Cat# E153A, Promega, Madison, WI). Cell lysates (20 µl) were incubated with 100 µl of luciferase assay buffer (20 mM Tricine pH 7.8, 1.1 mM MgCO3, 4 mM MgSO4, 0.1 mM EDTA, 0.27 mM coenzyme A, 0.67 mM luciferin, 33 mM DTT and 0.6 mM ATP). The intensity of the luminescence was measured using a Lumat LB 9507 luminometer (Berthold Technologies, Oak Ridges, TN). Conditions were performed in triplicate unless otherwise noted in the figure legend. Bar graphs display the mean fold change for each condition with error bars representing the standard error of the mean (S.E.M.).

### Confocal microscopy

HeLa cells cultured in 4 compartment CELLVIEW^TM^ glass-bottom dishes (Greiner Bio-One, Frickenhausen, Germany) were transfected at ~75% confluency with Lipofectamine 2000. Briefly, unless indicated in figure legends or the results section typical transfection conditions included 5 ng of GFP-tagged Dvl2, 10 ng of Frizzled, and empty vector to 250 ng per quadrant. Approximately 40 h post transfection HeLa cells were fixed with 4% paraformaldehyde (Electron Microscopy Sciences, Hatfield, PA) at room temperature after the initial removal of media. Fixed cells were washed thrice with Hank’s Balanced Salt Solution (HBSS) (Life Technologies, Carlsbad, CA). After the third wash, the quadrants were incubated with two drops of Image iT-FX signal enhancer (Molecular Probes, Eugene, Oregon) and rocked for 30–60 min at room temperature. Cells were then washed thrice with HBSS prior to the addition of V5 antibody (Novex, Carlsbad, CA) (1:1000) in 2% sterile filtered fraction V bovine serum albumin (BSA) (MP Biomedicals, Santa Ana, CA) containing HBSS solution at 4°C overnight. After the overnight incubation with the primary antibodies and three more washes the cells were incubated with Alexa Fluor 594 labeled secondary antibody (Molecular Probes, Eugene, Oregon) at room temperature in the dark for 90 min. Cells were washed 4 times again. The cells were then maintained in HBSS and stored at 4°C in the dark until Fluorescent and differential interference contrast (DIC) images were captured using a FluoView FV1000 confocal laser scanning microscope (Olympus, Tokyo, Japan) with a 60X oil immersion objective lens. Under permeabilization conditions the washing steps prior to the addition of the primary antibody included 0.2% Triton X-100 (Calbiochem, San Diego, CA) and 0.2% NP40 (Calbiochem, San Diego, CA).

### Receptor surface expression measured by IFA

Transfections for the immunofluorescence assay (IFA) were performed similarly to those of the functional assay with either 0.5 or 2 ng of receptor. 24 h after HEK293 cells plated on gelatin-coated black plate clear bottom 96 well assay plates (Corning Inc., Corning, NY) were transfected in triplicate they were fixed with 4% paraformaldehyde. Cells were washed with HBSS then blocked with 2% BSA. Cells were incubated with V5 antibody (Novex, Carlsbad, CA) at a 1:500 dilution, washed with HBSS and subsequently incubated with Alexa Fluor 594 donkey anti-mouse antibody (Molecular Probes, Eugene, OR) at a 1:1500 dilution. Cells were then washed with HBSS and fluorescence readings were taken with a SpectraMax M5 multimode plate reader (Molecular Devices, Sunnyvale, CA). Bar graphs display the mean relative fluorescence units (RFU) for each condition performed in triplicate unless otherwise noted in the figure legend with error bars representing the standard error of the mean (S.E.M.).

### Data analysis

To calculate the fold-change for Lef/Tcf-dependent transcriptional assays the ‘basal’ or ‘stimulated’ relative light units (RLU) values were first divided by the mean value from the 50 mM lithium chloride- treated wells of the same transfection condition, to determine the percent maximal stimulation. Those values were then divided by the percent maximal value of the mean basal condition in the wells transfected without Frizzled (hLRP5 alone) for “fold” change/activation. To test statistical significance between data, unpaired *t*-tests or one-way analysis of variance (ANOVA) followed by the Dunnett’s post test were conducted. *p* values <0.05 establish statistical significance.

## Results

**Figure 1:**
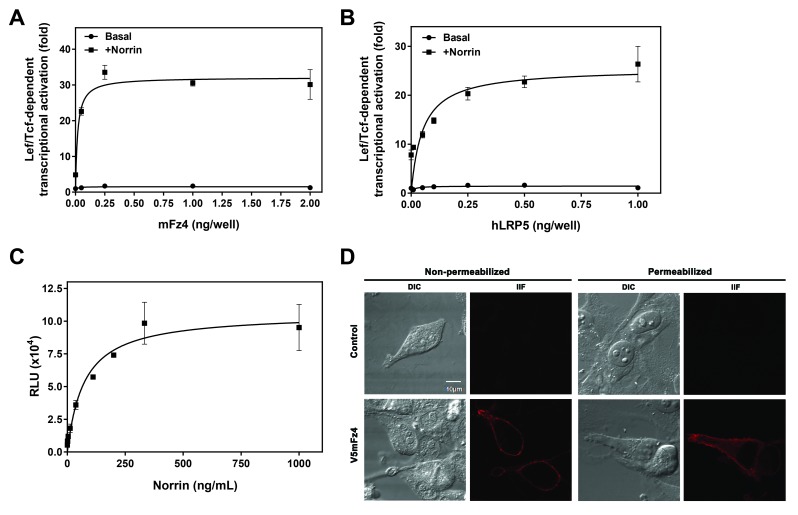
**Frizzled-4 Mediates Norrin induced Lef/Tcf-dependent transcriptional activation.** (A) Increasing concentrations of mouse Frizzled-4 (mFz4) were cotransfected into HEK293 cells with 1 ng of human hLRP5 construct (hLRP5) and stimulated with 200 ng/mL of recombinant Norrin and the Lef/Tcf-dependent luciferase enzyme induction was quantified as described in Materials and Methods with the exception that the basal values were determined for each condition in duplicate. (B) Increasing concentrations of hLRP5 coreceptor were cotransfected into HEK293 cells with 2 ng of mFz4 and stimulated with 200 ng/mL of recombinant Norrin and the Lef/Tcf-dependent luciferase enzyme induction was quantified with the exception that the basal values were determined for each condition in duplicate. (C) HEK293 cells cotransfected with 0.25 ng of mFz4 and 1 ng hLRP5 were stimulated with increasing concentrations of recombinant Norrin and the Lef/Tcf-dependent luciferase enzyme induction was quantified. (D) Transiently transfected V5-tagged mFz4 (V5mFz4) in HeLa cells were detected by means of indirect immunofluorescence (IIF) in the absence of or following a cell permeabilization step using a V5 primary antibody.

The ability of ectopically-expressed mouse Frizzled-4 (mFz4) to mediate Norrin-dependent beta-catenin stabilization was monitored by stimulating HEK293 cells transfected with increasing amounts of mFz4 and a fixed concentration of hLRP5 (1 ng per well of a 96-well plate) and recombinant Norrin (200 ng/mL). Between 0.25 and 2 ng of receptor DNA per well resulted in near maximal stimulation (Figure 1A). Interestingly, increasing the surface expression levels of receptor by transfecting higher amounts of receptor DNA per well (up to 15 ng of receptor DNA per well) eventually inhibited Norrin-mediated activation (Additional File 1A). Receptor expression increased in a concentration dependent manner (Additional File 1B). A total of 2 ng DNA per well of receptor DNA was employed to determine the optimal amount of hLRP5 DNA to cotransfect with the mFz4 (Figure 1B). Increasing hLRP5 transfection levels up to 0.5 ng resulted in a subtle (~1.5X) increase in basal Lef/Tcf-reporter activity (Figure 1B). Overexpressing LRP5 augmented Norrin-induced mFz4-mediated canonical activation. The synergism between mFz4 and hLRP5 in the presence of Norrin occurred at 1 ng hLRP5 DNA per well. Titrating in recombinant Norrin led to a maximal increase in Lef/Tcf dependent transcriptional activation when 0.25 ng of mFz4 and 1 ng of hLRP5 were employed at ~200 ng/mL of recombinant Norrin (Figure 1C). Thus 200 ng/mL of recombinant Norrin was deemed optimal under these conditions, as greater amounts of Norrin did not substantially increase transcriptional activation.

We checked for Frizzled localization in HeLa cells. Indirect immunofluorescence experiments were conducted utilizing a primary antibody specific for the V5 tag on the amino terminus of the mFz4 (V5mFz4). In non-permeabilized cells, V5mFz4 can be visualized on the cell surface. Permeabilization of cells revealed some additional receptors that are internal (Figure 1D).

**Figure 2:**
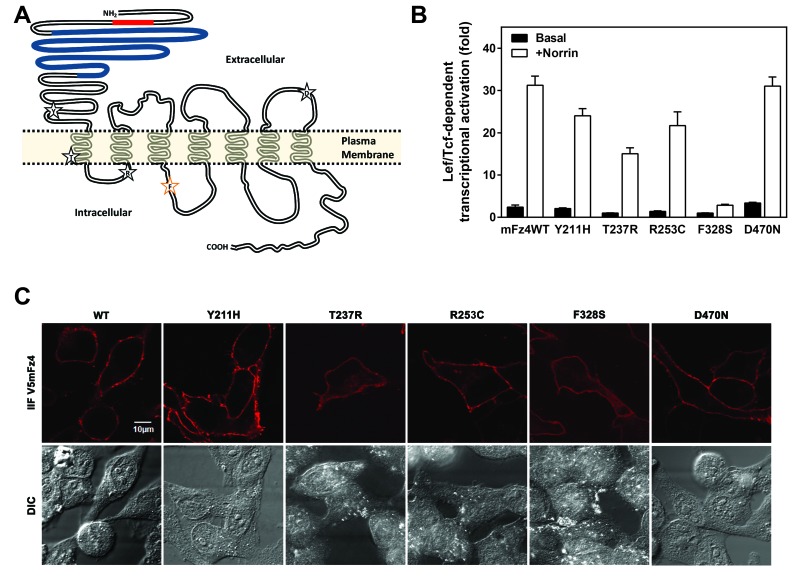
**Characterization of Frizzled-4 variants implicated in FEVR.** (A) A snake diagram of the V5-tagged mouse Frizzled-4 (V5mFz4) used in this study. The V5 epitope cloned into the amino terminus after the signal peptide is represented by a red shading. The putative CRD domain or ligand binding site is colored blue. The general localization of amino acids found to be mutated in certain FEVR patients that were investigated in this study are shown inside stars. F328 of iloop2 is displayed in an orange star. (B) V5mFz4 variants were overexpressed in HEK293 cells with hLRP5 and M50 reporter plasmid, stimulated with Norrin and Lef/Tcf-dependent luciferase activity was assayed as described. (C) IIF of nonpermeabilized HeLa cells transfected with V5mFz4 variants using a V5 primary antibody.

Human Fz4 mutations implicated in FEVR were engineered at the proper corresponding position on a V5mFz4 construct (Figure 2A). Human and mouse Fz4 sequence identity is ~97%. Intracellular loop 3 and the carboxy-terminus of Frizzleds have been shown to have a direct role in Dvl binding [18,19,20]. The cysteine-rich domain is also essential to Norrin binding [16]. We focused on mutations found in regions of the receptors outside of these regions. FEVR appears to result from disruption of normal ‘canonical’ activity [15]. We first determined the ability of mutant Fz4 to activate LEF/TCF-dependent transcription.

Signaling of the D470N Fz4 was similar to the WT Fz4. Mutations at Y211H, R253C, and T237R each reduced the ability of the Frizzled-4 to activate the canonical pathway, by approximately 25, 30, and 50%, respectively. Norrin-induced activation was most impaired in the F328S mFz4 (Figure 2B). Each of these mFz4 mutants exhibited expression on the cell surface (Figure 2C).

**Figure 3:**
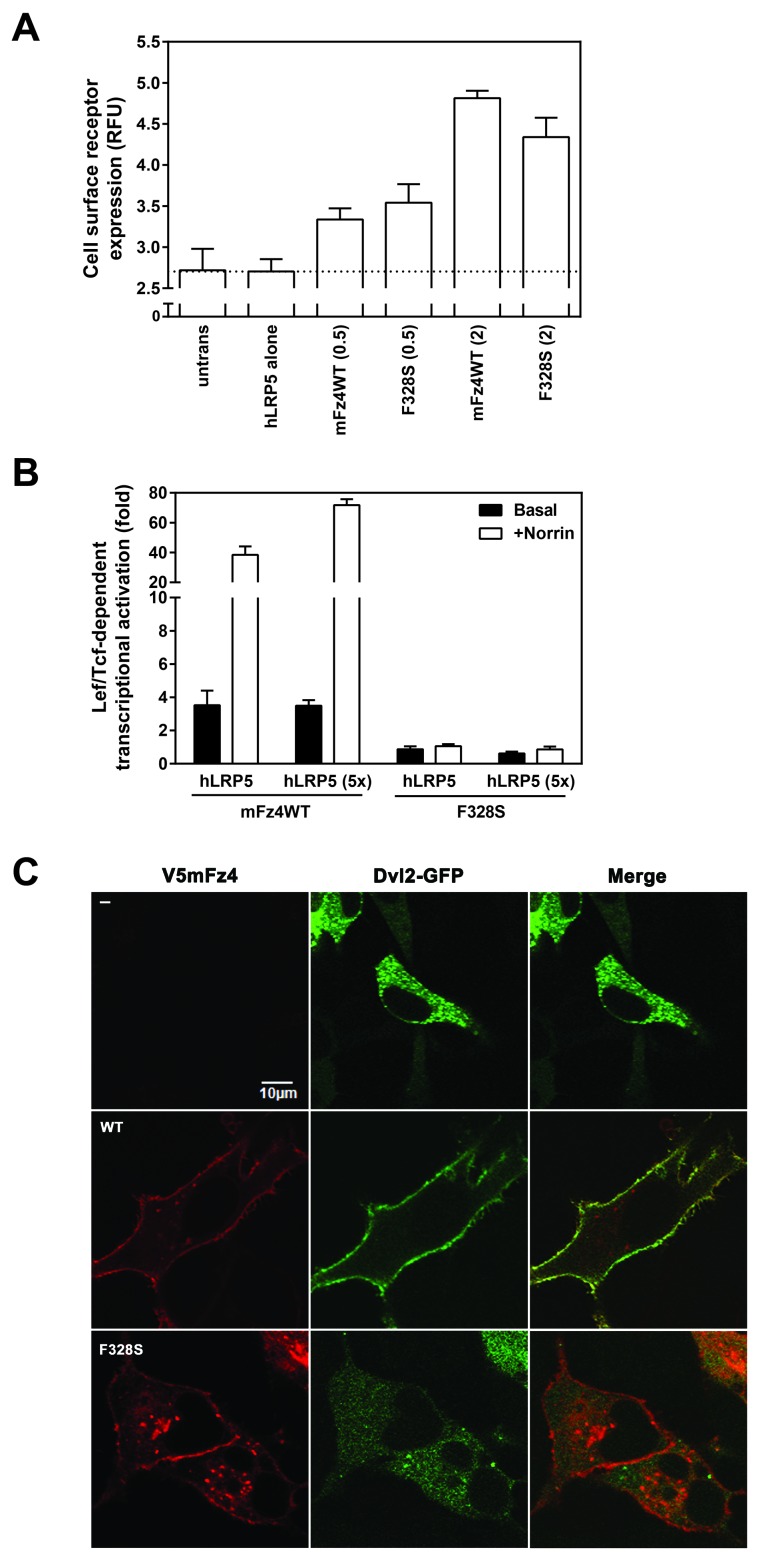
**F328S Frizzled-4 exhibits reduced Norrin-stimulation and Dvl2 recruitment.** (A) Cell surface receptor expression detected by IFA in HEK293 cells contransfected with the amount in ng of V5mFz4 variant shown in parentheses and 1 ng of hLRP5 per well. Following fixation the nonpermeabilized cells were incubated with V5 primary antibody followed by an incubation with a compatible alexaflour594 secondary antibody and subsequent fluorescence quantification on a multimode plate reader. The dotted line represents the background fluorescence level of the wells transfected with hLRP alone. (B) V5mFz4 WT and F328S were overexpressed in HEK293 cells and their ability to induce Lef/Tcf-dependent luciferase enzyme induction following the addition of recombinant Norrin was performed as described in the Materials and Methods with an exception being that the wells for the “5X” hLRP5 condition were transfected with 5 ng of hLRP5 instead of 1 ng. (C) Confocal images of permeabilized HeLa cells cotransfected with GFP-tagged Dvl2 and V5mFz4WT or F328S using a V5 primary antibody.

The cell surface expression of the F328S Fz4 was comparable to WTFz4. Cell surface localization was quantified in non-permeabilized HEK293 cells, transfected under the same conditions as those employed in the functional transcriptional activation (0.5 ng DNA per well of a 96-well plate) as well as that to the amount corresponding to the amount used in the Dvl2 recruitment assay (2 ng DNA per well of a 96-well plate). We made use of the V5 epitope on the amino terminus of Fz4 mutants and wild-type in combination with a modified enzyme-linked immunosorbent assay (ELISA). This IFA makes use of a fluorophore conjugated to the secondary antibody (in lieu of an enzyme). At 0.5 ng mutant DNA, the F328S Fz4 exhibited similar levels of surface expression, comparable to that of the WT receptor (Figure 3A). At higher concentrations of DNA, F328S Fz4 exhibited reduced surface expression. Thus the impaired signaling of F328S Fz4 is not due to an inability of the receptor to traffic to the cell surface. Reporter assays then were performed using five-fold more of hLRP5 coreceptor DNA to see if the reduction in F328S Fz4 signaling reflects a decreased ability to interact with the coreceptor (Figure 3B). Increasing the amount of hLRP5 coreceptor DNA did not rectify the impaired activation mediated by the F328S Fz4. Therefore, overexpression of LRP5 cannot overcome the impaired canonical signaling mediated by F328S Fz4.

Expression of Fz4 in HEK293 cells increases recruitment of Dvl2 to the cell surface in a ligand-independent manner [21]. Frizzled-Dishevelled interaction is critical to canonical signaling [22]. We analyzed the ability of Fz4 WT and F328S to recruit Dvl2 at the cell surface. Fz4 WT expression provoked Dvl2 localization to the cell surface whereas F328S expression did not (Figure 3C). Inspection of a field of cells coexpressing both Fz4 WT and Dvl2 demonstrated robust Dvl2 recruitment to the cell surface in all cases. Inspection of a field of cells coexpressing both Fz4 F328S and Dvl2 showed no such Dvl2 cell surface colocalization or clear partial Dvl2 recruitment to the cell surface. A five-fold greater amount of F328S plasmid DNA (50 ng plasmid DNA/quadrant) did not properly localize Dvl3 to the cell surface (data not shown).

**Figure 4:**
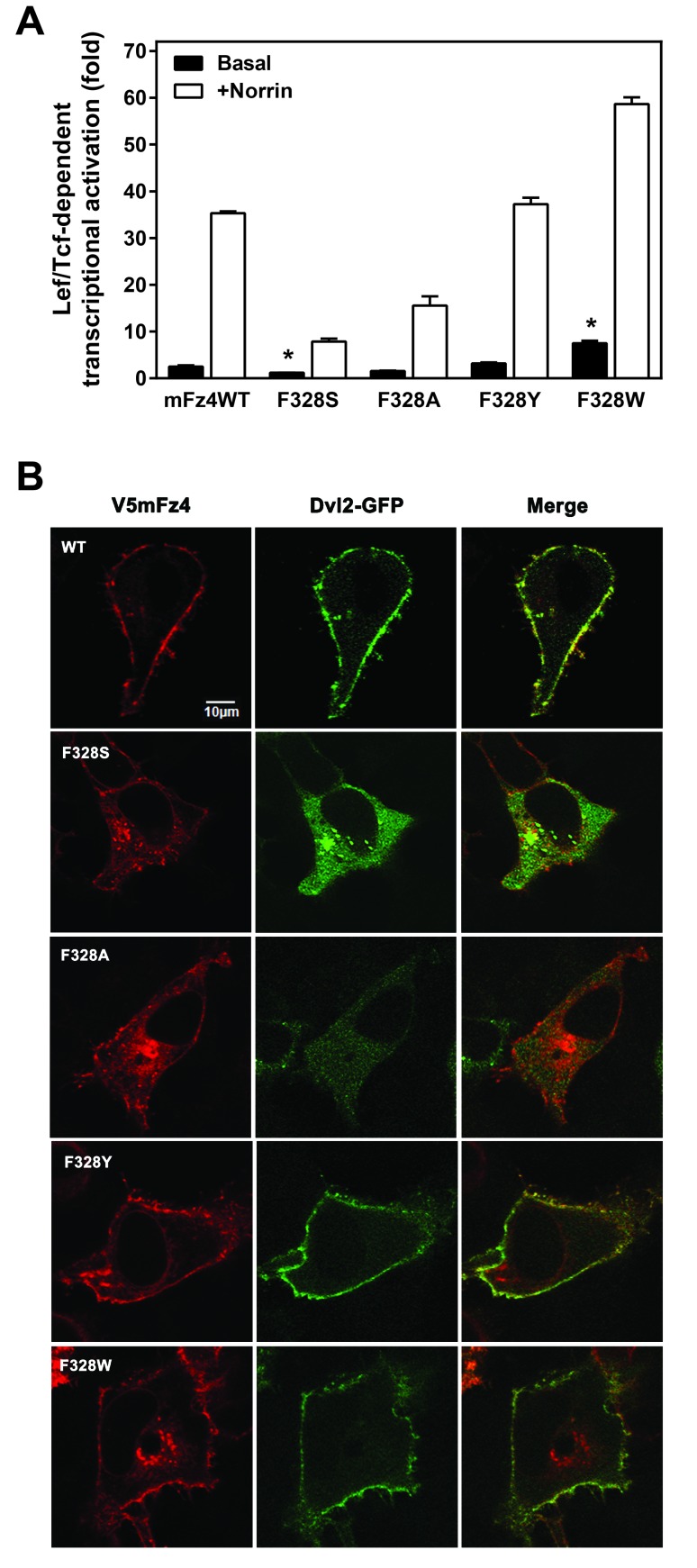
**Aromaticity is required at position F328 for normal Dvl2 recruitment and canonical activation.** (A) A series of V5mFz4 variants with substitutions at F328 were overexpressed in HEK293 cells with hLRP5 and M50 reporter plasmid, stimulated with Norrin and Lef/Tcf-dependent luciferase activity assayed. Statistically significant differences between basal value transcriptional activation of the variants compared to that of the WT receptors as determined by one-way analysis of variance (ANOVA) followed by the Dunnett’s post hoc test is indicated with an asterisk (*). (B) Confocal images of permeabilized HeLa cells cotransfected with GFP-tagged Dvl2 and V5mFz4WT or F328S using a V5 primary antibody.

A new series of mutations of Fz4 were generated at position 328. The F328A Fz4 displayed a reduced ability to activate the canonical pathway, much like the F328S mutant (Figure 4A). Interestingly, substituting tyrosine-for-phenylalanine had no detectable effect on Frizzled signaling. Substituting position 328 with a tryptophan residue yielded a Fz4 mutant that exhibited a modest increase in basal activation. The cell surface expression of F328W Fz4 was not greater than that of the WT (Additional file 1B).

The F328S and F328A Fz4s that exhibited the impaired Norrin-induced activation failed to recruit Dvl2 (Figure 4B). The F328Y and F328W Fz4, in constrast, recruited Dvl2 to the cell membrane in a normal manner.

**Figure 5:**
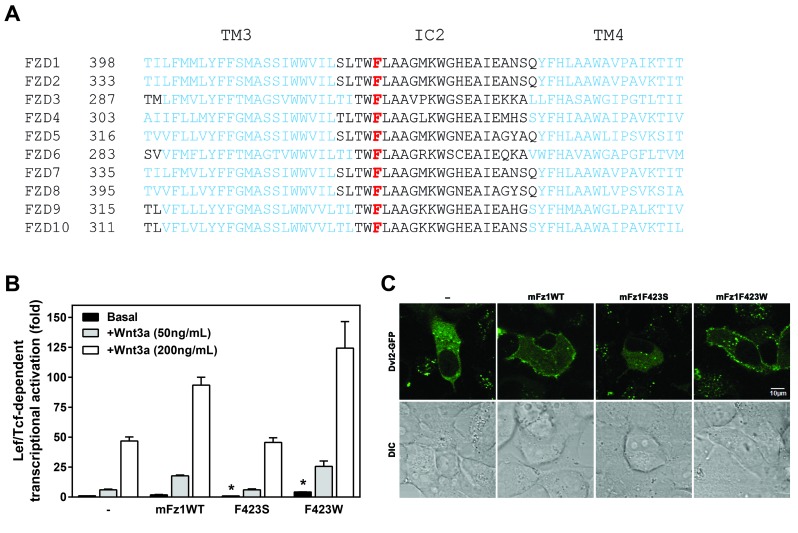
**Intracellular Loop 2 may have a conserved role in enabling a Frizzled-Dvl2 Interaction.** (A) A sequence alignment (adapted from an alignment generated at http://www.uniprot.org/) shows the aligned sequences of the intracellular loop 2 (IC2) regions of the 10 Frizzleds and the flanking transmembrane domain three (TM3) and four (TM4) displayed in blue. The phenylalanine residues at the corresponding position to F328 of mouse Frizzled-4 are indicated in red. (B) Frizzled 1 variants with mutations corresponding to Frizzled-4 F328S and F328W were overexpressed in HEK293 cells with hLRP5 and M50 reporter plasmid, and Lef/Tcf-dependent luciferase activity assay was performed as described in the Materials and Methods with an exception being that the wells were stimulated with recombinant Wnt3a in lieu of Norrin. Statistically significant differences between basal value transcriptional activation of the variants compared to that of the WT receptors as determined by an ANOVA analysis followed by the Dunnett’s post hoc test is indicated with an asterisk (*). (C) Confocal images of HeLa cells transfected with 10 ng GFP-tagged Dvl2 with or without Fz1 variants were obtained as described in the Materials and Methods.

The residue corresponding to F328 of Fz4 is completely conserved in each of the other 9 mammalian Frizzleds (Figure 5A). We generated mutations analogous to mFz4 F328S and F328W in the mouse Frizzled-1 (Fz1). F423S and F423W Fz1 were engineered and expressed. The ability of these Fz1s to signal Wnt3a-induced Lef/Tcf-dependent transcriptional activation in HEK293 cells was explored (Figure 5B). F423S Fz1 overexpression failed to enhance Wnt3a-induced Lef/Tcf-dependent transcriptional activation. The F423W mutation was able to increase the basal and the Wnt3a-induced transcriptional activation compared to that of the WT. As shown with Fz4, Fz1 also recruits Dvl2 to the cell surface (Figure 5C). Mutating F423 to serine in Fz1 (corresponding to F328S in Fz4) resulted in an inability for the Frizzled to recruit Dvl2 to the cell surface. Substitution of Fz1 F423 with tryptophan in contrast, displayed similar Dvl2 localization as WT.

## Discussion

In this study mutations of Fz4 associated with clinical manifestations in FEVR were investigated. One of the mutant Fz4s, F328S, demonstrated an impaired ability to activate LEF/TCF-dependent transcription. This mutant Wnt receptor was shown to traffic to the cell surface. Most interestingly, this Fz4 mutant was unable to recruit Dvl2.

Frizzleds demonstrate some degree of redundancy. In the absence of the other, Frizzled-2 or Frizzled-7 can mediate embryonic palate closure [14]. Norrin has been suggested to operate solely through activating Fz4 [16]. The possibility of another Frizzled masking the phenotype of defective Norrin-induced signaling seems unlikely. The discovery of leucine-rich repeat-containing, G-protein-coupled receptor 4 (LGR4) exhibiting high affinity Norrin binding [23] introduces a possibility of a non-Frizzled G protein-coupled receptor mediating its biology.

Dvl consists of three structured domains including a DIX domain critical for homo-oligomerization [24], and DEP and PDZ domains that may directly interact with cytoplasmic regions of Frizzleds. The carboxy-terminus of Fz4 displays the KTXXXW motif (where X is any residue) at the transmembrane domain 7 proximal region of the tail. This motif appears to have a critical role in the recruitment of Dvl to the cell membrane [25]. An interaction between a peptide encoding a portion of the Frizzled-7 carboxy-terminus including the KTXXXW motif and the PDZ domain of Dvl1 has been reported [18]. Peptides based on the intracellular loop three (iloop3) and carboxy terminus of Frizzled-5 are capable of binding various regions of Dvl1. These observations point to the interesting possibility that iloop3 of the Frizzleds may also be critical to docking of Dvl [19].

In this study we interrogated upon the possibility of iloop2 recruiting Dvl2. Supporting such a role is the prediction that transmembrane domain 3 proximal region of iloop2 (consisting of the amino acid sequence TLTWF) docks the PDZ domain of Dvl1, Dvl2, and Dvl3 [26]. Substituting the phenylalanine at the carboxy-terminus of the peptide with serine might be predicted to preclude the Dvl2-iloop2 docking. Five-mer peptides TLTWF, TLTWS, and TLTWW were tested in HeLa cells expressing Dvl2 and Fz4 and displayed no ability to act as dominant negative peptides, although reasons for this negative results could be many (data not shown).

The high degree of sequence homology between the PDZ, DEP, and DIX domains of the three isoforms of Dvl found in the mammalian genome does not preclude selective interaction of Dvl with Frizzleds. In NIH 3T3 cells, Fz4 docks Dvl1 and Dvl3, but apparently not Dvl2 [13]. Purified peptides encoding the carboxy-terminal domain of Fz4 support the notion that the carboxy-terminus of Fz4 has a higher affinity for the PDZ domain of Dvl2 than that of Dvl1 or Dvl3 [20].

Mutation of iloop2 of Fz4 is shown to alter Dvl2 recruitment. iloop2 of Fz4 may modulate interactions between the Frizzled and another signaling component such as LRP5/6 although Dvl has been shown to be phosphorylated in a Frizzled-dependent manner in the absence of coreceptor [27]. Therefore an inability of the receptor to activate LRP5/6 likely would not preclude Dvl recruitment.

## Conclusion

The F328S Fz4 mutation in iloop2 implicated in FEVR and related retinopathies is shown to display a reduced ability to activate Tcf/Lef transcription in response to Norrin and an inability to recruit Dvl2 at the cell surface. Mutational analysis of the corresponding residue of Frizzled-1 also resulted in a receptor incapable of recruiting Dvl2. We conclude that the iloop2 of Frizzleds may well have a direct (or indirect) role in Dvl localization and responsibility for clinical outcomes associated with FEVR and related retinopathies.

## Abbreviations

ANOVA: analysis of variance; BRB: blood retina barrier; BSA: bovine serum albumin; DIC: differential interference contrast; Dvl: Dishevelled; ELISA: enzyme-linked immunosorbent assay; FEVR: Familial exudative vitreoretinopathy; Fz4: Frizzled-4; GFP: green fluorescent protein; HBSS: Hank’s Balanced Salt Solution; HEK293: Human embryonic kidney; IFA: immunofluorescence assay; IIF: indirect immunofluorescence; iloop2: intracellular loop 2; iloop3: intracellular loop 3; LGR4: leucine-rich repeat-containing, G-protein-coupled receptor 4; LRP5: low-density lipoprotein receptor-related protein 5; M50: Super8xTOPFlash; mFz1: mouse Frizzled-1; mFz4: mouse Frizzled-4; RFU: relative fluorescence units; RLU: relative light units; S.E.M.: standard error of the mean; TM3: transmembrane domain three; TM4: transmembrane domain four; TSPAN12: transmembrane 4 superfamily member 12; WT: wild-type; ZNF408: Zinc finger protein 408

## Additional File

**Additional File 1: Frizzled-4 overexpression can inhibit Norrin-induced Lef/Tcf-dependent transcriptional activation.** HEK293 cells were transfected with 38.5 ng of empty vector, 1 ng hLRP5, 10 ng of M50, and the amount of V5-tagged Frizzled-4 variant shown (between 0 and 15 ng per well of a 96-well plate). Approximately 24 h post transfection the cells were either stimulated with Norrin or LiCl to monitor Lef/Tcf-dependent luciferase enzyme activity (**A**) or the cells were fixed with paraformaldehyde and V5 primary antibody was used in an IFA to determine relative receptor surface expression levels (**B**). The dotted line represents the mean background level of the wells transfected with hLRP5 alone.

## Competing Interests

The authors declare they have no competing interests.
